# Protective Effect of Octylmethoxycinnamate against UV-Induced Photoaging in Hairless Mouse via the Regulation of Matrix Metalloproteinases

**DOI:** 10.3390/ijms19071836

**Published:** 2018-06-22

**Authors:** So Young Kim, Santosh Lamichhane, Jung-Hun Ju, Jaesuk Yun

**Affiliations:** 1 National Institute of Food and Drug Safety Evaluation (NIFDS), Ministry of Food and Drug Safety (MFDS), OHTAC 187, Osongsaengmyong 2-ro, Cheongju-si, Chungbuk 28159, Korea; soyouki@hanmail.net (S.Y.K.); jjheun@korea.kr (J.-H.J.); 2 College of Pharmacy, Wonkwang University, Iksandaero 460, Iksan, Jeonbuk 54538, Korea; lakki38786@gmail.com

**Keywords:** UV, photoaging, sunscreen agent, octylmethoxycinnamate, matrix metalloproteinase

## Abstract

Ultraviolet (UV) irradiation damages skin and produces symptoms of photoaging, such as thickening, rough texture, wrinkles, and pigmentation. However, the cellular and molecular mechanisms underlying photoaging induced by chronic UV irradiation are not yet fully understood. Matrix metalloproteinases (MMPs) have been reported to be involved in the response to UV irradiation. In this study, we examined the effects of the sunscreen agent Octylmethoxycinnamate (OMC) on photoaging of the skin induced by chronic UV exposure in hairless albino Crl:SKH1-*Hrhr* (SKH-1) mice. We demonstrated that the expression of MMPs was elevated by UV irradiation, whereas the topical application of OMC inhibited the upregulation of MMPs. Furthermore, UV-induced wrinkle formation was decreased by OMC treatment. These results suggest that OMC is a potential agent for the prevention and treatment of skin photoaging.

## 1. Introduction

The physical symptoms of skin aging induced by ultraviolet (UV) irradiation in human skin include thickening, rough texture, coarse wrinkles, and mottled pigmentation [1]. Type I collagen is the most abundant structural protein in skin connective tissue and is synthesized primarily by fibroblasts residing within skin connective tissue (dermis). Fibroblasts secrete a soluble precursor, type I procollagen, which is proteolytically processed to form insoluble collagen fibers. The disorganization, fragmentation, and dispersion of collagen bundles are prominent features in photodamaged human skin [2]. Various cells, such as keratinocytes, fibroblasts, and inflammatory cells, produce matrix metalloproteinases (MMPs) under UV irradiation. It has been suggested that MMPs induce excessive matrix degradation and contribute substantially to the connective tissue damage that occurs during photoaging [3]. MMPs are endopeptidases that may participate in the degradation of different macromolecular components of the extracellular matrix and the basement membrane, including collagen [4]. UV exposure induced an increase in the expression of MMP-1, -3, and -9 in the normal human epidermis and MMP-13 in human dermal fibroblasts [5]. These results indicate that the repeated degradation of collagen by UV-induced MMPs may lead to its deficiency in photodamaged skin. These properties make MMPs an attractive target for the development of anti-photoaging agents.

It has been reported that Octylmethoxycinnamate (OMC) has an inhibitory effect on the formation of UV-induced wrinkles in hairless albino Crl:SKH1-*Hrhr* (SKH-1) mice [6]. Despite many investigations into the effects of OMC in UV-induced damage, the ameliorative effects of OMC on photoaging induced by chronic UV irradiation are not fully understood. Therefore, in the present study, we evaluated the protective effects of OMC against UV-induced photoaging in *SKH-1* hairless mice, focusing on the regulation of MMPs.

## 2. Results

**Figure 1:**
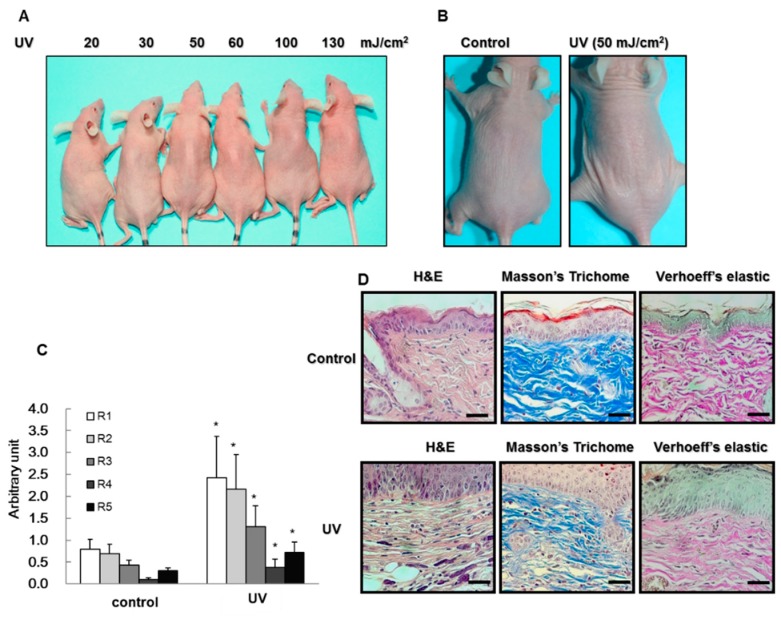
(**A**,**B**) Measurement of minimal erythema dose (MED). The intensity of ultraviolet (UV) irradiation that induced minimal erythema in the dorsal skin of hairless albino Crl:SKH1-*Hrhr* (SKH-*1*) mice was 50 mJ/cm^2^; (**C**) Wrinkle formation induced by UV irradiation. UV irradiation increased R1, R2, R3, R4, and R5 values. R1 is the distance between the highest peak and the lowest value. R3 is the average of the five maximum distances; R1 and R2 are the largest values of those five maximum distances; R5 indicates the average (arithmetic) roughness; R4 indicates smoothness depth. The data are expressed as the mean ± S.E. (*n* = 5); * *p* < 0.05 vs. control (Student’s *t*-test); (**D**) Hematoxylin and Eosin (H&E) staining, Masson-Trichrome staining, and Verhoeff’s elastic staining of the dorsal skin of UV-irradiated SKH-1 hairless mice. The UV-irradiated group showed inflammation, decreased collagen fibers, and decreased elastin expression. Scale bar = 20 μm.

To determine the mechanisms involved in UV-induced photoaging, hairless albino Crl:SKH1-*Hrhr* (SKH-1) mice were irradiated with UV (20, 30, 50, 60, 100, and 130 mJ/cm^2^) for 15 weeks. At the end of period, the minimal erythema dose (MED) of UV irradiation was determined as 50 mJ/cm^2^ (Figure 1A,B). The wrinkle depth of the skin surface was measured by using light transmission from a silicone replica. UV irradiation induced a severe increase in total roughness (the distance between the highest peak and the lowest value, R1), maximum roughness (the largest value of the five maximum distances, R2), average roughness (average of the five maximum distances, R3), smoothness depth (R4), and arithmetic average roughness (R5) (Figure 1C). Akin photoaging is caused by chronic UV exposure, which results in a change in the dermal level of collagen and elastin. The histopathological changes in the skin of *SKH-1* hairless mice were analyzed by staining with Masson’s trichrome, in which collagen, keratin, and the nuclei are stained blue, red, and black, respectively. The results revealed that collagen formation in the skin was decreased by UV irradiation (Figure 1D). We then performed elastin staining to detect the production of elastin in the skin. Elastin levels in the skin were also reduced by UV irradiation (Figure 1D).

**Figure 2:**
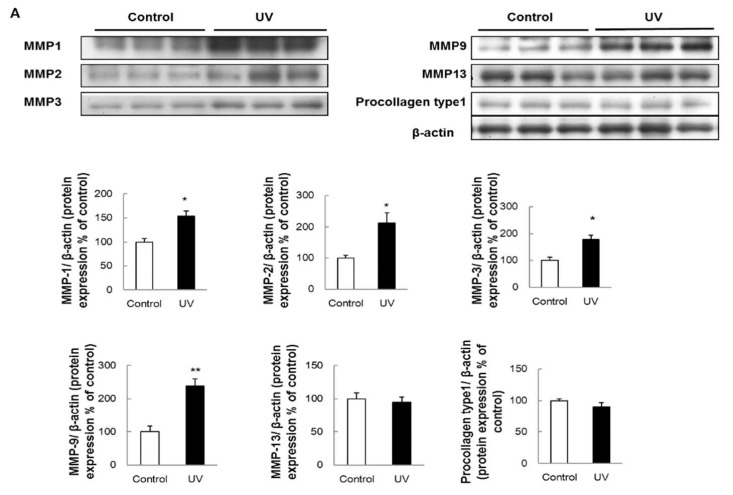

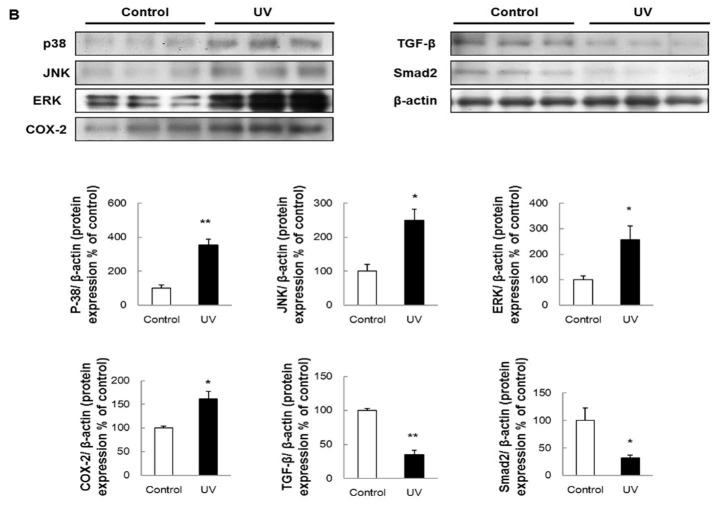
Effects of UV irradiation on the expression of (**A**) Matrix metalloproteinases (MMPs) MMP-1, MMP-2, MMP-3, MMP-9, MMP-13, procollagen type 1, and (**B**) p38, JNK, ERK, COX-2, TGF-β, and Smad2 in SKH-1 hairless mice. Representative figures show the densitometric analysis of the immunoblotting results of the control and UV-irradiated group, quantified by using ImageJ software. The data are expressed as the mean ± S.E. (*n* = 3); * *p* < 0.05, ** p < 0.01 vs. control (Student’s *t*-test).

Furthermore, skin biopsies were obtained and subjected to western blotting for detecting various key proteins involved in the biological functions of the extracellular matrix (MMP-1, MMP-2, MMP-3, MMP-9, MMP-13, and procollagen type I), Mitogen-activated protein kinase (MAPK) signaling pathways p38, c-Jun N-terminal kinase (JNK), and Extracellular signal-regulated kinase (ERK), inflammation Cyclooxygenase-2 (COX-2), and immunity and defense Transforming growth factor beta (TGF-β) and Smad2 (Figure 2A,B). The results showed that UV upregulated the expression of MMP-1, MMP-2, MMP-3, MMP-9, p38, JNK, ERK, and COX-2. However, the expression of TGF-β and Smad2 were reduced by UV irradiation.

**Figure 3:**
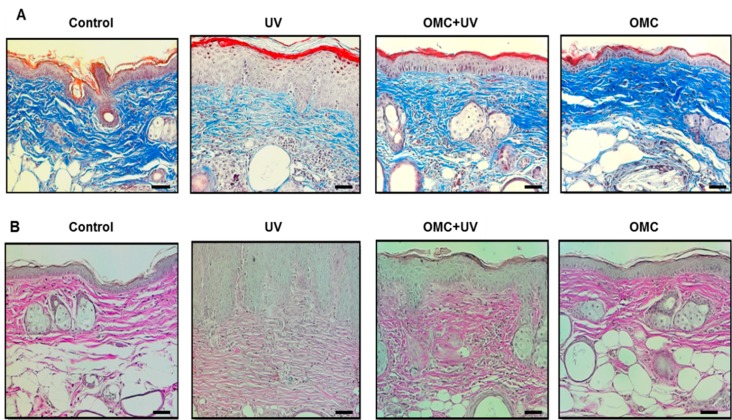
Effects of Octylmethoxycinnamate (OMC) on collagen fibers and elastin expression in UV-irradiated SKH-1 hairless mice. (**A**) After a 15-week application of OMC, histological observation of collagen fibers in photoaged SKH-1 hairless mice skin with Masson’s Trichrome stain was performed; (**B**) After a 15-week application of OMC, histological observation of elastin in photoaged SKH-1 hairless mice skin was performed with Verhoeff’s stain. Representative figures show that OMC rescued the UV-increased decrease in collagen fibers and elastin expression. Scale bar = 20 μm.

To examine the photoprotective effect of OMC on the histopathologic changes induced by chronic UV exposure, SKH-1 hairless mice were treated with UV (50 mJ/cm^2^) for 15 weeks. In the OMC-treated + UV-irradiated group, collagen levels were improved in comparison with those in the UV-irradiated group, whereas collagen staining in the OMC-treated group was not different from that in the vehicle-control group (Figure 3A). The UV-induced reduction of elastin was abrogated by pretreatment with OMC (Figure 3B).

**Figure 4:**
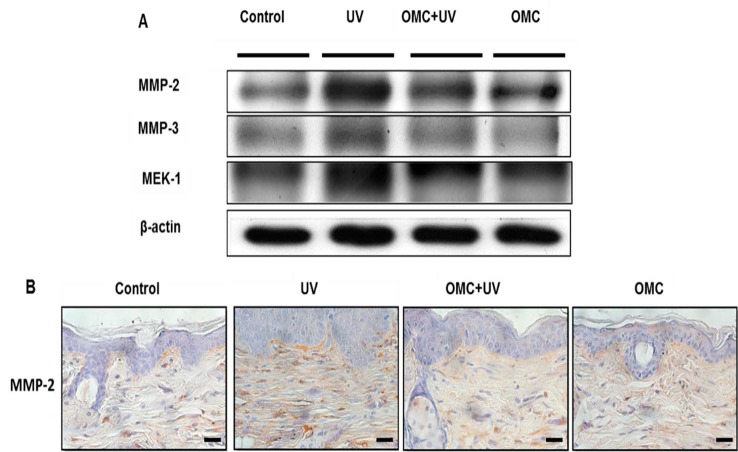
Effects of OMC on MMPs and MEK-1 expression in UV-irradiated SKH-1 hairless mice. (**A**) Immunoblotting results showed that UV-induced upregulation of MMP-2, MMP-3, and MEK-1 expression was inhibited by OMC; (**B**) The immunohistochemical results showed that UV-induced upregulation of MMP-2 expression was inhibited by OMC. Scale bar = 20 μm.

MMP production is well established in photodamaged human skin. Therefore, we examined the effects of OMC on MMP proteins in UV-irradiated SKH-1 hairless mouse by using immunohistochemistry and western blotting (Figure 4A,B). The expression of MMP-2 was elevated markedly upon UV irradiation; in contrast, OMC attenuated the upregulation of UV-induced MMP-2 expression (Figure 4A,B). These results indicated that OMC may function as an anti-photoaging agent through the interruption of MMP-dependent signaling pathways.

**Figure 5:**
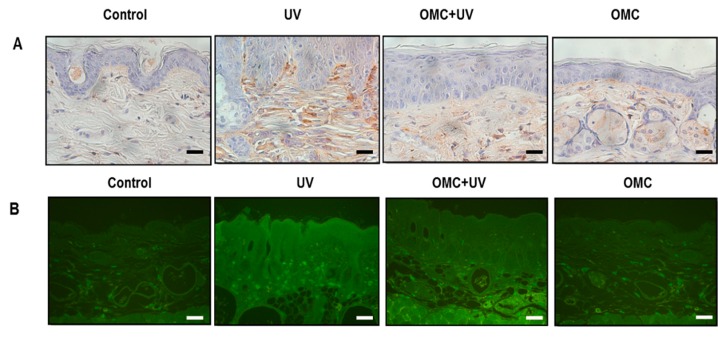

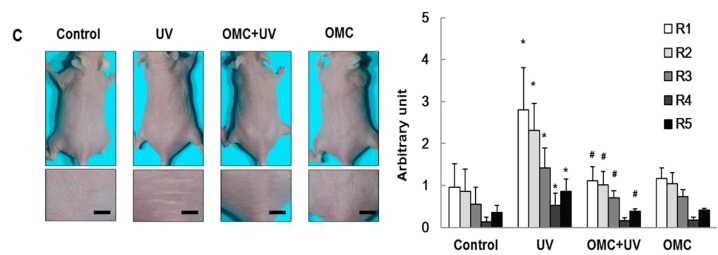
Effects of OMC on Survivin and Daxx expression in the skin of UV-irradiated SKH-1 hairless mice. Immunoblotting results showed that UV-induced upregulation of (**A**) Survivin and (**B**) Daxx was inhibited by OMC. Scale bar = 20 μm; (**C**) Effects of OMC on wrinkle formation in UV-irradiated SKH-1 hairless mice. Representative photographs show wrinkles of rats in the control, UV, OMC + UV, and OMC groups. Scale bar = 5 mm. UV irradiation increased the values of R1, R2, R3, R4, and R5. OMC treatment inhibited wrinkle formation in the OMC + UV group; * *p* < 0.05 vs. control and ^#^ *p* < 0.05 vs. UV (two-way ANOVA followed by Bonferroni post-hoc tests).

As UV-induced MMP upregulation is associated with apoptosis, we examined the effects of OMC on the expression of proteins related to apoptosis, such as (Figure 5A) and Daxx (Figure 5B). These results showed that OMC modulated UV-induced upregulation of apoptosis-related protein expression. Next, we examined the effects of OMC on UV-induced wrinkle formation in the dorsal skin of SKH-1 hairless mouse. UV irradiation markedly induced wrinkle formation, which was abrogated by the topical application of OMC on the dorsal skin of hairless mice (Figure 5C).

## 3. Discussion

UV irradiation interferes with the preservation of the extracellular matrix and participates significantly in the development of premature skin aging [7,8]. In a previous study, we identified the expression changes in genes associated with the effects of UV radiation through the analysis of the photoaging-related genes MMP-13, laminin (β), procollagen, ccl3, ccl4, cxcl10, ccl9, p16, and caspase 9 in the skin of UV-irradiated SKH-1 hairless mouse [6]. OMC is one of the most frequently used UV filters in sunscreens to protect the skin against damages from the noxious influence of UV radiation [9]. However, the inhibitory actions of OMC in photoaging have not yet been defined.

It has been demonstrated that UV irradiation triggers the synthesis of MMPs in human skin in vivo [10,11]. MMP-mediated collagen destruction accounts for a large proportion of the connective tissue damage that occurs in photodamaged skin. MMPs exhibit a wide specificity towards a variety of ECM components and participate both directly and indirectly in several MMP activation cascades. These properties make MMPs an excellent target for the pharmacological and cosmetic development of anti-photodamage agents. In this study, we attempted to elucidate the molecular mechanisms underlying UV irradiation-induced MMP synthesis and its amelioration by OMC treatment. Our data suggest that OMC not only ameliorated ultraviolet (UV)-induced decrease of collagen and elastin, but also mitigated UVB-induced MMP expression in the SKH-1 hairless mouse. As collagen deficiency in chronic UV exposure may result from the increased deterioration of collagen by UV-induced MMPs, our results suggest that OMC treatment may prevent UV-induced connective tissue damage through the inhibition of UV-induced MMP expression.

Several studies are in progress to evaluate the effects of UV irradiation-induced activation of multiple cell surface cytokines, growth factor receptors, and Mitogen-activated protein MAP kinase pathways, and rapid induction of transcription factor Activator protein 1 (AP-1) activity [11,12]. It is reasonable to speculate that the expression of mRNA and protein of the AP-1 family members c-Jun and c-Fos in human skin fibroblasts was induced by UV irradiation in our experiments [13,14]. AP-1 regulates the transcription of several MMPs, including MMP-1, MMP-3, and MMP-9. Additionally, increasing evidence suggests that UV irradiation induces c-Jun, a vital component of the transcription factor AP-1, in keratinocytes and fibroblasts of normal human skin in vivo. c-Jun directly interacts with activated Smad3 in the nucleus and prevents its binding to target genes [15]. Recent studies have indicated that the MAP kinase signal transduction pathways play a crucial role in the regulation of a variety of cellular functions [16,17,18], including cell growth [19,20], MMP expression [21], and type I procollagen synthesis [22,23]. In this study, we found that OMC inhibited UV-induced activation of MEK-1 (MAPK/ERK kinase). 

Anti-apoptotic genes such as Survivin and Daxx have also been associated with UV-induced photoaging [6]. Survivin, an IAP (inhibitor of apoptosis protein), plays a role in oncogenesis, such as in oral cancer [24,25]. Daxx is a Fas-binding protein that enhances Fas-mediated apoptosis and activates the Jun N-terminal kinase (JNK) pathway [26]. It was report that the inhibition of apoptosis by tissue inhibitor of metalloproteinase-1 (TIMP-1) was mediated by the inhibition of MMPs in hepatic cells [27,28]. In this study, OMC inhibited UV-induced expression of Survivin and Daxx in SKH-1 hairless mice, which suggests a possible biological role of OMC as an anti-apoptotic agent. Thus, these results suggest that OMC is a potential agent for the prevention and treatment of skin photoaging.

## 4. Materials and Methods 

### 4.1. Chemicals and Reagents

OMC was purchased from Sigma Chemical Co. (St. Louis, MO, USA). OMC was dissolved in a 4:1 mixture of acetone/olive oil (AOO). Other routine chemicals were purchased from Sigma Chemical Co. (St. Louis, MO, USA), unless otherwise specified. 

### 4.2. Animals and UV Light Source

Female hairless albino Crl:SKH1-*Hrhr* (SKH-1) mice (6–7 weeks old, pathogen-free) were purchased from Charles River Laboratory (Wilmington, MA, USA) and were housed in an animal facility with full AAALAC (Association for Assessment and Accreditation of Laboratory Animal Care International, NITR-07, 1 January 2007) accreditation. Four mice were kept in each cage; the cages were maintained under controlled conditions (temperature, 23 ± 2 °C; relative humidity, 55 ± 10%; 12 h light cycle, lights on from 08:00 to 20:00), and were given a solid diet and tap water ad libitum. For UV irradiation, the mice were housed in specially designed cages where they were held in a divider separated by Plexiglas. The distance from the light source to the target skin was 23 cm for all UV irradiation experiments [29]. OMC is a *Food and Drug Administration* (FDA)-approved UV filter commonly used in commercial sunscreens. It is used as a UVB (280–320 nm) filter, even though the low-energy tail of its absorption spectrum extends into the UVA (320–400 nm) spectrum [30]. UV irradiation was performed with RMX-3W lighter (Dong Sung Lab Tech., Seoul, Korea) using a F40M UVB lamp (maximum emission wavelength, 312 nm; Vilber Lourmat, Marne-La-Vallee, France). The irradiation intensity was monitored by using a VLX-3W radiometer (Vilbert-Lourmat) equipped with VLX-312 (UVB) sensors.

### 4.3. Animal and Treatment

Female SKH-1 hairless mice, housed and maintained in the conditions described above, were used. To determine the MED of UV in SKH-1 hairless mice, the mice were divided into six groups and exposed to UV irradiation at 20, 30, 50, 60, 100, and 130 mJ/cm^2^ for 15 weeks. The mice were irradiated three times per week as follows: Week 1, 1 MED; Week 2, 1–2 MED, Week 3, 2–3 MED, Week 4, 3–4 MED; Weeks 5–15, 3–4 MED. Other mice were divided into four groups containing eight animals each. Each treatment (200 µL) was applied topically to the skin of mice three times per week for 15 weeks; 30 min after each application, the mice were exposed to UV irradiation. The mice in the first group received a topical application of 200 µL AOO and served as the vehicle control. The mice in the second group received a topical treatment of 200 µL AOO followed by UV irradiation (50 mJ/cm^2^) of their dorsal skin. The mice in the third group received a topical application of 200 µL 7.5% OMC followed by UV irradiation (50 mJ/cm^2^) of their dorsal skin. The animals in the fourth group received a topical application of 200 µL 7.5% OMC in the absence of UV irradiation. The mice were sacrificed 6 h after the final irradiation in Week 15, and the skins were collected.

### 4.4. Measurement of the Wrinkle Depth on the Skin Surface

The wrinkle depth was measured by light transmission from a silicone replica (Skin-Visiometer^®^ SV 600, CK electronic GmbH, *Köln*, Germany). The images of the replica of the dorsal area were captured with silicone polymer (SILFLO impression material, Flexico, Colchester, UK) and a CCD camera (SDC-45, Samsung, Suwon, South Korea). The topography of the skin surface was evaluated for total roughness (the distance between the highest peak and the lowest value, R1), maximum roughness (the largest value of the five maximum distances, R2), average roughness (the average of the five maximum distances, R3), smoothness depth (R4), and arithmetic average roughness (R5).

### 4.5. Preparation of Skin Lysates

The skins for biopsies were collected and pooled from each mouse in each treatment group. The subcutaneous tissue was removed by using a shaving scalpel, each skin was washed several times with 0.05 M Tris-HCl buffer (pH 7.4, containing 0.15 M NaCl), and then the skin samples were homogenized using a Polytron homogenizer in ice-cold lysis buffer containing protease inhibitors (Tris-HCl: 50 mM, pH 7.4; 0.1% NP-40; 150 mM NaCl; 0.02% sodium azide; 1 mM Ethylenediaminetetraacetic acid (EDTA); 1 mM Phenylmethylsulfonyl fluoride (PMSF); 1 µg/mL aprotinin, and 1 mM NaF). The homogenates were centrifuged at 14,000× *g* for 20 min at 4 °C; the resulting supernatants were used immediately or stored at −80 °C.

### 4.6. Immunoblot Analysis

The samples’ protein content was determined by using a Bio-Rad DC protein assay kit (Hercules, CA, USA) in accordance with the manufacturer’s protocol. For immunoblotting analysis, 25 µg protein was resolved on a 12% Tris-glycine polyacrylamide gel and then electrophoretically transferred onto nitrocellulose membranes. Non-specific binding to the membranes was blocked using 5% nonfat dry milk, and the membranes were probed using primary antibodies against MMP1 (1:1000), MMP2 (1:1500), MMP3 (1:1000), MMP9 (1:1000), MMP13 (1:1000), procollagen type 1 (1:500), p38 (1:1000), JNK (1:1000), ERK (1:1000), COX-2 (1:1000), TGF-β (1:1000), Smad2 (1:1000), and β-actin (1:5000), followed by incubation with horseradish peroxidase-conjugated secondary antibodies (Amersham Life Science Inc., Arlington Heights, IL, USA). The proteins were detected by chemiluminescence, using an ECL kit (Amersham Life Science) and autoradiography with X-ray films (Amersham Life Science).

### 4.7. Immunostainings

The avidin–biotin–peroxidase complex (ABC) technique was used for immunohistochemical staining. Before staining, 4 μm paraffin sections were deparaffinized in xylene and rehydrated in a graded alcohol series. Endogenous peroxidase activity was inhibited by incubation in 3% H_2_O_2_ in methanol. The slides were first incubated with normal horse serum and with normal goat serum to block nonspecific binding and sequentially incubated with the primary antibodies at 4 °C overnight and anti-mouse/rabbit biotinylated bridging antibodies (dilution 1:200) for 30 min. The sections were then washed and incubated with a standard avidin–biotin complex (ABC; DakoCytomation, Glostrup, Denmark) for 30 min. Antibody binding was revealed by using H_2_O_2_ as a substrate and diaminobenzidine as the chromogen, with counterstaining performed with hematoxylin. For collagen and elastin staining, Goldner’s modified Masson-Trichrome staining and Verhoeff’s staining were used, respectively.

### 4.8. Statistical Analysis

All data are presented as the mean ± S.E. The data were analyzed using Student’s *t*-test and two-way Analysis of Variance (ANOVA), followed by Bonferroni post-hoc tests using SigmaPlot 13 software (SigmaPlot, Chicago, IL, USA). Probabilities less than 5% (*p* < 0.05) were considered statistically significant.
